# Potential vectors of bluetongue virus in high altitude areas of Yunnan Province, China

**DOI:** 10.1186/s13071-019-3736-9

**Published:** 2019-10-04

**Authors:** Ying Liang Duan, Glenn Bellis, Le Li, Hua Chun Li, Hai Sheng Miao, Mei Ling Kou, De Fang Liao, Zheng Wang, Lin Gao, Ji Zhong Li

**Affiliations:** 1 Yunnan Tropical and Subtropical Animal Virus Diseases Laboratory, Yunnan Animal Science and Veterinary Institute, Kunming, Yunnan Province China; 2 Research Institute for the Environment and Livelihoods, Charles Darwin University, Darwin, NT Australia; 3 Zhongdian Animal Disease Control Center, Shangri-La, Yunnan Province China

**Keywords:** *Culicoides*, High altitude, Bluetongue virus vector, *C. tainanus*, *C. nielamensis*, *C. obsoletus*

## Abstract

**Background:**

Bluetongue disease of ruminants is a typical insect-borne disease caused by bluetongue virus (BTV) of the genus *Orbivirus* (family *Reoviridae*) and transmitted by some species of *Culicoides* (Diptera: Ceratopogonidae). Recently, the detection of BTV in yaks in high altitude meadows of the Shangri-La district of Yunnan Province, China, prompted an investigation of the *Culicoides* fauna as potential vectors of BTV.

**Methods:**

A total of 806 *Culicoides* midges were collected by light trapping at three sites at altitudes ranging from 1800 to 3300 m. The species were identified based on morphology and the DNA sequences of cytochrome *c* oxidase subunit 1 (*cox*1). PCR and quantitative PCR following reverse transcription were used to test for the presence of BTV RNA in *Culicoides* spp. A phylogenetic analysis was used to analyze the *cox*1 sequences of some specimens.

**Results:**

Four species dominated these collections and *cox*1 barcoding revealed that at least two of these appear to belong to species new to science. *Culicoides tainanus* and a cryptic species morphologically similar to *C. tainanus* dominated low altitude valley collections while *C. nielamensis* was the most abundant species in the high-altitude meadow. A species related to *C. obsoletus* occurred at all altitudes but did not dominate any of the collections. BTV RT-qPCR analysis detected BTV RNA in two specimens of *C. tainanus*, in one specimen closely related to *C. tainanus* and in one specimen closely related to *C. obsoletus* by barcode sequencing.

**Conclusions:**

This study suggests that BTV in high altitude areas of Yunnan is being transmitted by three species of *Culicoides*, two of which appear to be new to science. This research may be useful in improving understanding of the effects of global warming on arboviral disease epidemiology and further study is important in research into disease control and prevention.

## Background

Bluetongue disease of ruminants is caused by bluetongue virus (BTV) of the genus *Orbivirus* (family *Reoviridae*), which is a notifiable disease to the World Organization for Animal Health (OIE, Office International des Epizooties). The virus is transmitted by species of *Culicoides* [1]. There are 1368 extant species of *Culicoides* [2] but only about 30 of these have been associated with transmission of BTV [1, 3–6]. Bluetongue virus has mainly been reported in tropical and subtropical areas of Africa, Australia, North and South America, Asia and the Middle East with seasonal outbreaks reported from Mediterranean countries and Europe [7]. The unprecedented northward spread of BTV into Europe in the early 2000s has been linked to the effects of climate change and its influence on both the distribution and vector capacity of local *Culicoides* populations [3, 8].

BTV has been isolated from many parts of China since 1979 [9–11] with at least 14 BTV serotypes identified by serological investigation and virus isolation [10, 12]. Some species of *Culicoides* have been considered as vectors of BTV, including *C. oxystoma* Kieffer (as *C. schultzei* Enderlein), *C. peregrinus* Kieffer, *C. arakawai* (Arakawa) and *C. circumscriptus* Kieffer [13]; however, solid evidence to support these assertions is lacking. A number of proven and suspected vector species are present in China including *C. actoni* Smith, *C. brevitarsis* Kieffer, *C. fulvus* Sen & Das Gupta, *C. obsoletus* (Meigen) and *C. wadai* Kitaoka, although some of these species are actually a complex of species and therefore require confirmation [14]. Recently, Ma et al. [15] reported the presence of BTV in yaks and sheep on the Tibetan Plateau, western China, at an altitude of over 3000 m but little is known of the species of midges living at such high altitudes or their potential to transmit BTV.

Yunnan Province is located in the southwest of China and shares a border with Laos, Myanmar and Vietnam. While the southern tropical and sub tropical districts of Yunnan are endemic for BTV [12], the north of Yunnan is mostly cold, high altitude mountains and river valleys, and there is little information on the prevalence of BTV or other arboviruses in this area.

**Fig. 1:**
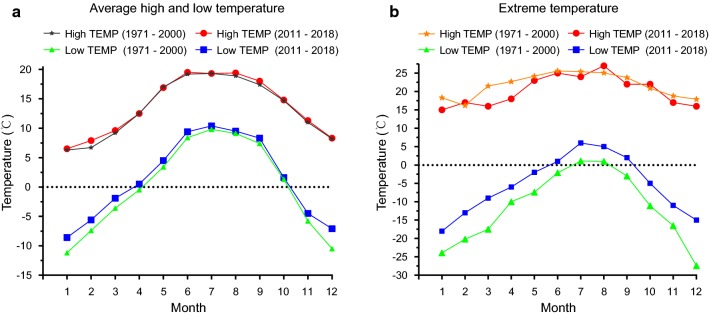
Historical maximum and minimum temperature records in Shangri-La District, Yunnan, China. **a** Average maximum and minimum monthly temperatures (TEMP) between 2011 and 2018 and between 1971 and 2000. **b** Monthly maximum and minimum temperatures between 2011 and 2018 and between 1971 and 2000

Shangri-La district is located in the northwest of Yunnan, next to the Tibetan Plateau, with an area of 11,613 km^2^. The Shangri-La district consists of relatively warm river valleys and cold highland meadows. The river valleys are located about 1900 m above sea level along the Changjiang River and contain many small tributary streams. The highland meadows average about 3450 m above sea level and are covered by snow for almost four months of the year. There is, however, some evidence that this extreme climate is becoming milder as historical weather records indicate that the average monthly minimum temperatures between 2011 and 2018 are about 1 °C higher than that of the years 1971 to 2000 (Fig. 1a). Furthermore, the monthly minimum temperatures between 2011 and 2018 were milder than between 1971 and 2000 (Fig. 1b) [16]. The impact of such changes is difficult to predict but could result in increased activity of *Culicoides* spp. and associated arboviruses in this region.

During an investigation between 2014 and 2017, an average of 5% of yaks living in a 3290 m altitude highland meadow of Shangri-La were found to have BTV antibodies [17]. In a nearby river valley at a lower altitude, a 35–65% seroprevalence of BTV and a BTV-21 strain was isolated from some goat farms [17] indicating that BTV is prevalent at these altitudes. There are scarce data on the species diversity and abundance of *Culicoides* spp. at high altitudes in Asia so it is unclear if any local species can survive the cold and long winters experienced in these areas. Some midge species are, however, able to survive at high altitudes in South America [18] and Europe [19] so it is likely that at least some Asian species are also able to survive in the Shangri-La area. Also unclear is whether the BTV found in yaks in the highland meadows is endemic or introduced seasonally by the immigration of infected midges from the warmer river valleys.

This paper provides a brief account of the diversity and abundance of *Culicoides* spp. on livestock farms in the Shangri-La district of Yunnan Province, China and the screening of the most common species for the presence of BTV RNA using real-time quantitative PCR.

## Methods

### *Culicoides* spp. collection

*Culicoides* were collected from farms at three different sites. The first site was on a farm (27°03′36″N, 100°04′12″E) with 4 cattle and 24 goats near Jinjiang village along the Changjiang River, at an altitude of 1800 m. The second site was on a farm (28°06′00″N, 99°25′12″E) with 2 cattle and 85 goats near Nixi village, located in a small tributary river valley named Longchi at an altitude of 2200 m. The third site was on a farm (27°30′36″N, 99°48′36″E) with 15 yaks and 41 goats near Xiaozhongdian village, in the middle of a highland meadow area at an altitude of 3300 m. Mains-powered UV light traps (LTS-M02, Wuhan Lucky Star Medical Treatment Technology Co., Wuhan, China) were used for trapping *Culicoides*. Two light traps were placed close (10 m) together at each site for one night and ran from 16:00 h to 9:00 h the following day. The insects were collected directly into 70% ethanol and stored at 4 °C. All collections were made in the period 8th to 15th August 2017.

### Morphological identification

Insect specimens were sorted into morphospecies based on gross morphology and wing pattern using keys and illustrations of Yu et al. [20]. Parous and gravid females free from visible blood were selected based on abdominal pigmentation [21] and contents and submitted individually for nucleic acid extraction. Following digestion, representative specimens were mounted onto microscope slides using the technique of Bellis et al. [22] and identified morphologically using the keys and descriptions of Yu et al. [20].

### DNA and RNA extraction

Whole midges were digested non-destructively following the methods of Bellis et al. [22]. Briefly, insects were immersed in 300 µl of DXT tissue digestion buffer (Qiagen, Hilden, Germany) incorporating 1% DX digestion buffer additive (Qiagen) and incubated at 40 °C overnight. Following digestion, the insect cadaver was removed from the digestion lysate and preserved in 70% ethanol for subsequent morphological examination as described above. From the lysate, 220 µl was used for DNA extraction and the remaining 50 µl was used for RNA extraction. The RNA was extracted using a MagMAX™-96 viral RNA Isolation kit (Am1836; Ambion, Austin, TX, USA) following the manufacturer’s directions and using a MagMAX™ Express-96 machine (Ambion). The DNA was extracted using a DNeasy Blood & Tissue kit (#69560; Qiagen) following the manufacturer’s directions. The final DNA elution of 120 µl and RNA of 50 µl were stored at − 20 °C.

### Pan BTV serotype RT-qPCR

The primers BTVF-MH, BTVR-MH and probe BTVP-MH for real-time quantitative polymerase chain reaction (qPCR) described by Hofmann et al. [23] were used to detect the *NS3* gene of BTV in insect specimens. Reverse transcription quantitative PCR (RT-qPCR) was performed on a Fast7500 Realtime PCR machine (Applied Biosystems, Carlsbad, CA, USA) using an AgPath-ID™ one step RT-PCR Kit (Ambion). The reaction was performed as described by the manufacturer on a 2 µl RNA sample in a total volume of 20 µl, using PCR master mix with 6 mM MgCl_2_, 1 μM of each PCR primer and 0.2 μM of each probe. The RT-qPCR program consisted of the following: 10 min reverse transcription at 45 °C; 10 min denaturation at 95 °C; 45 cycles of 15 s denaturation at 95 °C, 45 s annealing and extension at 65 °C. Fluorescence was measured at the end of each annealing step. Only reactions with Cq values of 25 or lower were regarded as indicative of replication of BTV in the midge [4, 24, 25].

### *cox*1 gene amplification

Two specimens morphologically referable to *C. nielamensis* Liu & Deng, 19 specimens morphologically referable to *C. tainanus* Kieffer and 9 specimens morphologically referable to *C. obsoletus* (Meigen) including the 4 specimens found to be infected with BTV, were processed and sequenced for cytochrome *c* oxidase subunit 1 (*cox*1). Primers BC1culicFm and JerR2m, and techniques described by Bellis et al. [22], were used to amplify the *cox*1 barcode region. Resultant sequences were compared with published data on Barcode of Life Data System (BOLD) and the National Center for Biotechnology Information (NCBI).

### Sequence analysis

The PCR products of *cox*1 (692 bp) genes from *Culicoides* spp. were sent to a local company for sequencing using the Sanger method on an ABI3739XL machine (Kunming Shuoqing Biological Technology Company, Kunming, China).

*cox*1 sequences of representative *Culicoides* specimens and the most similar *Culicoides* spp. from NCBI data were analyzed by alignment using MEGA-X. Pairwise Kimura 2-parameter (K2P) distances among sequences were calculated and compared as a neighbor-joining (NJ) tree (bootstrap = 1000) using MEGA-X [26].

## Results

### *Culicoides* species

**Fig. 2:**
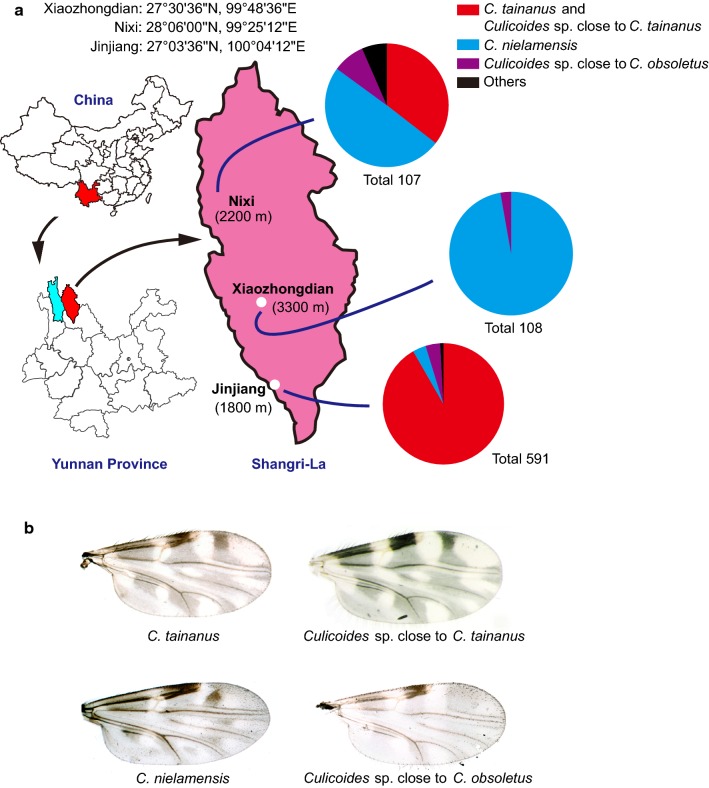
*Culicoides* spp. collection sites in Shangri-La, Yunnan, China. **a** Relative abundance of *Culicoides* spp. collected from three sites in Shangri-La. **b** Photographs of wings of the dominant species collected in Shangri-La, Yunnan, China

Weather conditions at the three sites were typical for the season. The temperatures in the afternoon at the collection sites were 28 °C (Jinjiang), 25 °C (Nixi) and 20 °C (Xiaozhongdian). The total number of midges collected at Jinjiang, Xiaozhongdian and Nixi was 591, 108 and 107, respectively. Specimens with morphology consistent with *C. tainanus*, *C. nielamensis* and *C. obsoletus* dominated collections, comprising 98% of all specimens collected (Fig. 2 and Table 1). Other species collected included a species similar to *C. punctatus* Latreille, another close to *C. sinanoensis* Tokunaga, and five species which remain unidentified due to lack of description in reference books (Table 1).

**Table 1:** Relative abundance of *Culicoides* species found in Jinjiang, Nixi and Xiaozhongdian, Yunnan Province, China

Town/village	Altitude (m)	Coordinates	Subgenus	Species	Relative abundance (%)
Xiaozhongdian	3300	27°30′36″N, 99°48′36″E	*Avaritia*	*C. nielamensis*	97.22 (105/108)
			*Avaritia*	*Culicoides* sp. close to *C*. *obsoletus*	2.78 (3/108)
Nixi	2200	28°06′00″N, 99°25′12″E	*Avaritia*	*C. nielamensis*	49.53 (53/107)
			*Avaritia*	*C. tainanus* and *Culicoides* sp. close to *C*. *tainanus*^a^	35.51 (38/107)
			*Avaritia*	*Culicoides* sp. close to *C*. *obsoletus*	8.41 (9/107)
			*Culicoides*	*Culicoides* sp. close to *C*. *punctatus*	1.87 (2/107)
			*Avaritia*	*Culicoides* sp. close to *C. tainanus*	1.87 (2/107)
			*Avaritia*	*Culicoides* sp. close to *C*. *sinanoensis*	0.93 (1/107)
			*Culicoides*	Unknown	0.93 (1/107)
			Unknown	Unknown	0.93 (1/107)
Jinjiang	1800	27°03′36″N, 100°04′12″E	*Avaritia*	*C. tainanus* and *Culicoides* sp. close to *C*. *tainanus*^a^	91.70 (542/591)
			*Avaritia*	*Culicoides* sp. close to *C*. *obsoletus*	3.89 (23/591)
			*Avaritia*	*C. nielamensis*	3.55 (21/591)
			*Hoffmania*	Unknown	0.51 (3/591)
			*Hoffmania*	Unknown	0.17 (1/591)
			Unknown	Unknown	0.17 (1/591)

^a^These two species cannot easily be distinguished morphologically so accurate counts of each species were not made. Based on *cox*1 analyses, the ratio of *C. tainanus* and *Culicoides* sp. close to *C*. *tainanus* in these collections was 7:12

**Fig. 3:**
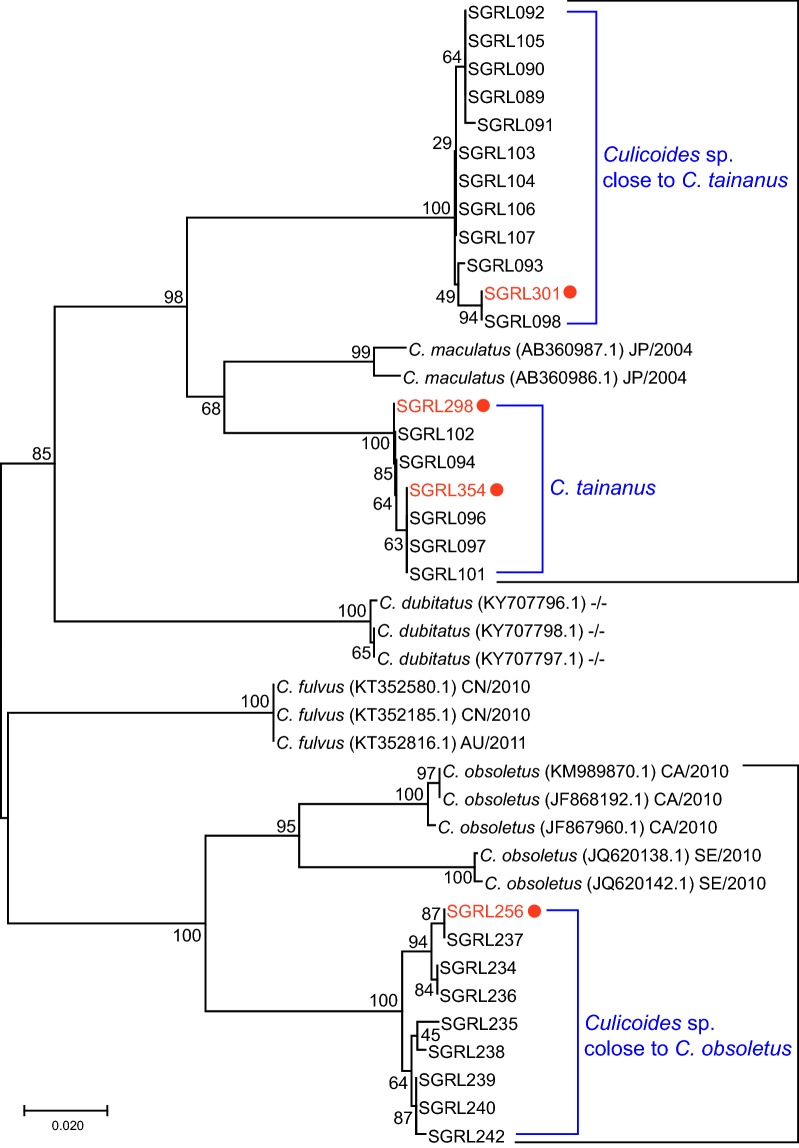
Neighbor-Joining tree for *Culicoides* spp. collected in Shangri-La based on the pairwise genetic distances among 41 mitochondrial *cox*1 sequences from specimens of *C. tainanus*, *Culicoides* sp. close to *C. tainanus* and *Culicoides* sp. close to *C. obsoletus* from Shangri-La and closest species matches from NCBI. Specimens from Shangri-La bear a number prefixed with “SGRL” (NCBI accession numbers MN513299- MN513326). Specimens containing BTV RNA are labelled by solid circles. For the specimens from NCBI, the country of collection and GenBank accession numbers are provided. *Country codes*: JP, Japan; CN, China; AU, Australia; CA, Canada; SE, Sweden; “-/-“indicates that no information on country of origin is available

*cox*1 sequences of 8 specimens morphologically identified as *C. obsoletus* (BOLD accession numbers SGRL234-SGRL240 and SGRL242) and 16 specimens morphologically identified as *C. tainanus* (BOLD accession numbers SGRL089-SGRL094, SGRL096-SGRL098 and SGRL101-SGRL107) were registered on BOLD. A neighbor-joining tree of these specimens (Fig. 3) revealed the presence of two clades of *C. tainanus*. One of these clades was 99.47% similar to *C. tainanus* (= *C. maculatus* Shiraki) sequences on BOLD while the other was only 90.12% similar to those specimens (Table 2). So far, we have been unable to distinguish specimens from these two clades morphologically, so we are tentatively calling this second species *Culicoides* sp. close to *C. tainanus*.

**Table 2:** Comparison of *cox*1 sequence data of the four dominant species of *Culicoides* collected in Shangri-La with publically available sequence data

Species	Morphological identification	Collection site	Best matches	
			BOLD	GenBank
1	*C. tainanus*	Nixi and Jinjiang	*C. tainanus* (99.47%) [unpublished]	*C. maculatus* (92.40%) [AB360987.1]
2	*Culicoides* sp. close to *C. tainanus*	Nixi and Jinjiang	*C. tainanus* (90.12%) [unpublished]	*C. maculatus* (89.61%) [AB360986.1]
3	*Culicoides* sp. close to *C. obsoletus*	Nixi, Jinjiang, Xiaozhongdian	*C. obsoletus* (92.12%) [unpublished]	*C. obsoletus* (90.11%) [JF867960.1]
4	*C. nielamensis*	Nixi, Jinjiang, Xiaozhongdian	No matched result	*C. minutissimus* (84.14%) [KJ767954.1]

*Note*: *C. maculatus* = *C. tainanus*

**Fig. 4:**
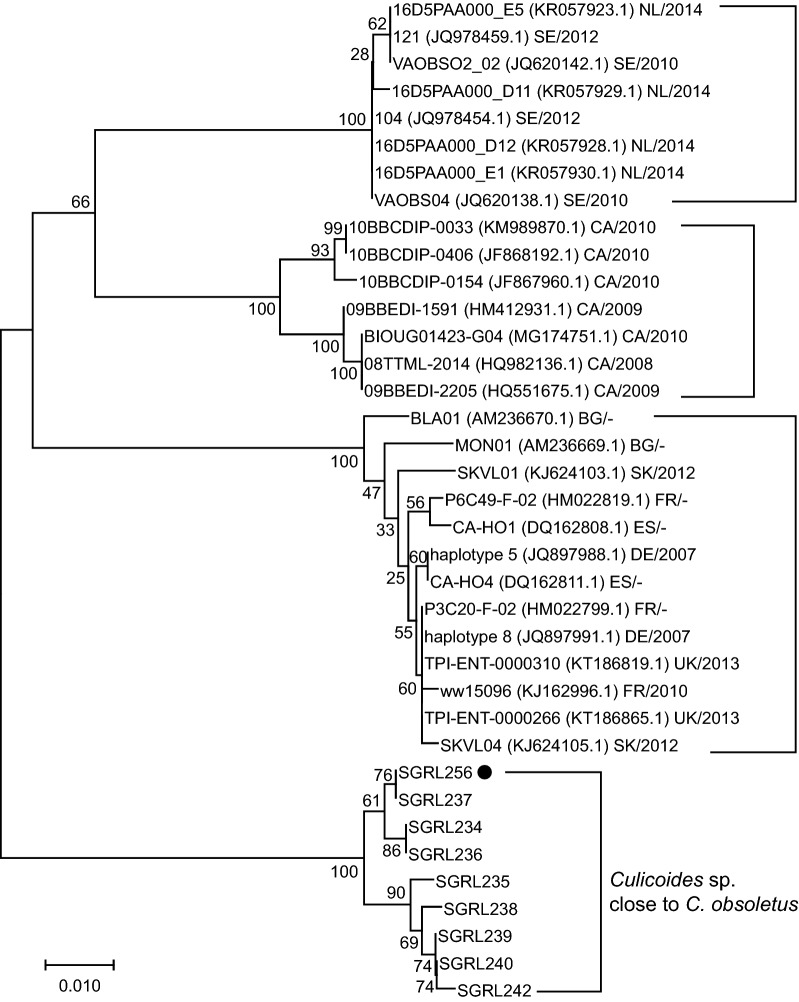
Neighbor-joining tree for *Culicoides* sp. close to *C. obsoletus* collected in Shangri-La based on the pairwise genetic distances among the mitochondrial *cox*1 sequences from 9 *Culicoides* sp. close to *C. obsoletus* from Shangri-La and 28 representative sequences for *C. obsoletus* from NCBI. Specimens from Shangri-La bear a number prefixed with “SGRL” (NCBI accession numbers MN513318–MN513326). Specimen containing BTV RNA is labelled by solid circle. For the specimens from NCBI, the isolates, GenBank accession numbers, country of collection and collection years are provided. *Country codes*: NL, Netherlands; SE, Sweden; CA, Canada; BG, Bulgaria; SK, Slovakia; FR, France; ES, Spain; DE, Germany; UK, United Kingdom; “–” indicates that no information of collection year is available

Similarly, we found no close matches for our specimens resembling *C. obsoletus* (Figs. 3, 4). The closest match available on NCBI or BOLD was for *C. obsoletus* from Canada, suggesting that our specimens are close to, but different from, true *C. obsoletus* and possibly represent a cryptic species.

No reference sequences in BOLD nor NCBI were close to our *cox*1 sequences from *C. nielamensis*; the nearest sequence was 84.14% similar and belonged to *C. minutissimus* Zett (Table 2).

The relative abundance of these four dominant species varied with altitude, *C. tainanus* and *Culicoides* sp. close to *C. tainanus* were only found at low and mid-altitudes, *C. nielamensis* was most abundant at high altitude and *Culicoides* sp. close to *C*. *obsoletus* was found at all altitudes. From high to low altitude, the relative abundance of *C. nielamensis* was 97.22, 49.53 and 3.55%, *C. tainanus* and *Culicoides* sp. close to *C. tainanus* was 0, 35.51 and 91.70% and *Culicoides* sp. close to *C. obsoletus* was 2.78, 8.4% and 3.89% (Fig. 2 and Table 1).

### BTV RT-qPCR of *Culicoides*

**Fig. 5:**
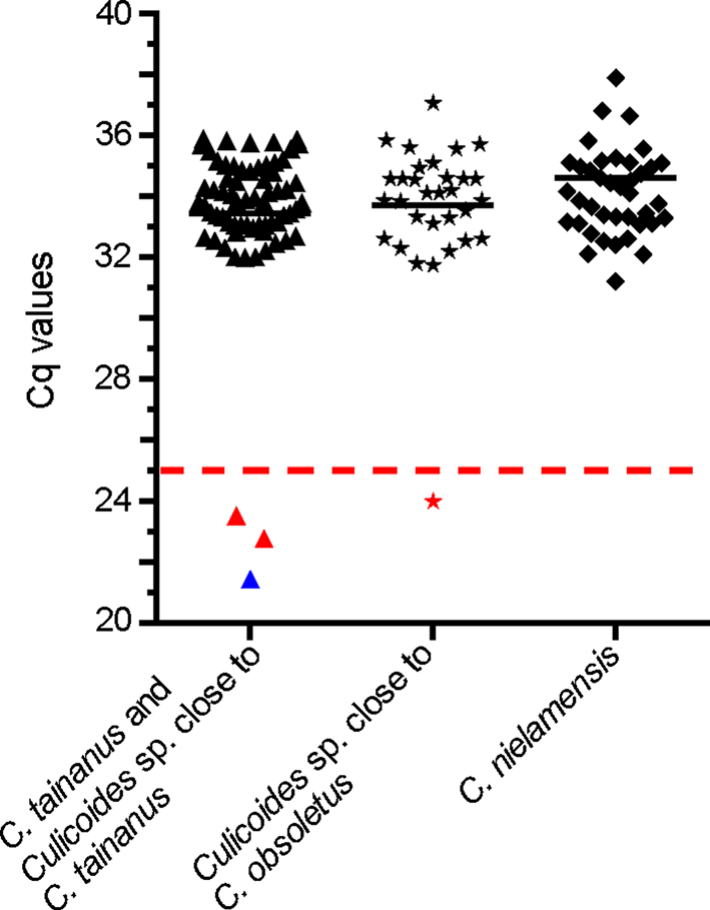
Identifying BTV vectors by qPCR. The BTV NS3 gene was detected by qPCR, and the 4 samples with Cq values of less than 25 are shown as red triangle (*C*. *tainanus*), blue triangle (*Culicoides* sp. close to *C. tainanus*) and red star (*Culicoides* sp. close to *C. obsoletus*)

A total of 150 specimens of *C. tainanus* and *Culicoides* sp. close to *C. tainanus*, 35 *Culicoides* sp. close to *C. obsoletus* and 80 *C. nielamensis* were analyzed for the presence of BTV. Many specimens of all species had detectable Cq values, including 70 *C. tainanus* and *Culicoides* sp. close to *C. tainanus*, 32 *Culicoides* sp. close to *C. obsoletus* and 42 *C. nielamensis*. Cq values exhibited a bimodal distribution with 4 specimens having values of 25 or less and the remainder of specimens with values between 30 and 35 (Fig. 5).

Cq values below 25 have been shown in both colony [24] and field-collected [4, 25] specimens to indicate that viral replication has occurred in the insect so the 4 specimens with Cq values less than 25 were considered infected with BTV. These 4 specimens were all collected from Nixi and were all parous females; 2 were identified as *C. tainanus*, 1 was *Culicoides* sp. close to *C. tainanus* and 1 was *Culicoides* sp. close to *C. obsoletus* (Fig. 3 and Table 3).

**Table 3:** Species, physiological age grade and Cq values of the 4 specimens identified as infected with BTV by quantitative PCR for the BTV Seg10 gene

Specimen number	Sex	Physiological age grade	Cq value	Species identification	
				Based on morphology	Based on *cox*1 barcode
SGRL256	Female	Parous	24.01	*C. obsoletus*	*Culicoides* sp. close to *C. obsoletus*
SGRL298	Female	Parous	22.79	*C. tainanus*	*C. tainanus*
SGRL301	Female	Parous	21.46	*C. tainanus*	*Culicoides* sp. close to *C. tainanus*
SGRL354	Female	Parous	23.54	*C. tainanus*	*C. tainanus*

*Note*: 806 *Culicoides* were collected, and only 4 out of 265 tested specimens had a Cq value lower than 25

## Discussion

To our knowledge, this is the first investigation of BTV vectors at high altitude areas of Yunnan and follows the detection of an average 5% BTV seroprevalence in yaks at 3290 m altitude of Jiantang and 35–65% BTV seroprevalence in goats at 2000–2800 m altitude of Nixi in Shangri-La [17]. Based on our limited data, the abundance of midges in this area is low compared to tropical or sub-tropical areas of Yunnan where over 10,000 midges can be collected after 12 hours trapping (our unpublished data).

The low prevalence of BTV observed in yaks at 3300 m could be due to a variety of factors, many of which are related to the impact of the harsh environment on vector biology, for example low vector abundance, low vector competence or short season of vector activity. The data from this brief study are not sufficient to allow firm conclusions about the absolute abundance of vector populations at different altitudes but some differences in midge diversity were evident between the three sites studied. The change in dominance from *C. tainanus* and *Culicoides* sp. close to *C. tainanus* at low altitude to *C. nielamensis* at high altitude is evident and coupled with the detection of BTV in *C. tainanus* and *Culicoides* sp. close to *C. tainanus* but not in *C. nielamensis* may be a contributing factor to the lowered BTV prevalence at high altitude.

*Culicoides* sp. close to *C. obsoletus* was present only in low numbers at all sites which may reduce its potential to act as a significant vector of BTV. Specimens from Shangri-La are morphologically similar to the European *C. obsoletus*, but significantly different in *cox*1 (about 9%). The Obsoletus group of *Culicoides* subgenus *Avaritia* has been shown to contain several species with very similar morphology and it is likely the species present in Shangri-La is a further cryptic species within this complex. Species from this complex are Holarctic so it is not surprising that populations are able to survive in high altitude sites such as Shangri-La. Further study on this group in Asia may reveal that the species present in Shangri-La is more widely distributed than currently known. Some species of the Obsoletus group have also been implicated in the transmission of BTV [6, 27, 28], so it is not surprising that a further member of this group may also be acting as a vector of BTV.

Recent work in Japan [29] found evidence that *C. tainanus* (= *C. maculatus* Shiraki) may be acting as a vector of BTV and this is supported by the detection of BTV in specimens from Shangri-La. The presence of a morphologically similar species in Asia that may also be a vector of BTV highlights the need for ongoing studies of the taxonomy of species in this region, particularly species belonging to the subgenus *Avaritia* of *Culicoides*. Integrative taxonomy using a combination of morphological and molecular analyses is currently the best method of establishing the identity of specimens [14] and should be applied to any studies of the vector status of species.

Specimens of *Culicoides* sp. close to *C. obsoletus*, *C. tainanus* and *Culicoides* sp. close to *C. tainanus* were found to contain BTV RNA in concentrations consistent to indicate virus had replicated in the insect thus fulfilling one of the criteria of these species being proven vectors of BTV [30, 31]. Consistent with the findings of previous studies [4, 24, 25], many specimens of midges from Shangri-La exhibited a positive response in the RT-PCR but the high Cq values from these specimens suggest either a non-specific response or a response due to remnant virus in the midge following a viraemic blood meal. The absence of specimens with Cq values between 25 and 30 supports the conclusion of Veronesi et al. [24] and van der Saag et al. [4, 25] that the virus has indeed multiplied in those midges with Cq values of less than 25.

None of the 80 specimens of *C. nielamensis* tested showed evidence of infection with BTV suggesting a prevalence of less than 1 in 80 (< 1.25%) compared to a prevalence of > 2.0% for *C. obsoletus*, *C. tainanus* and *Culicoides* sp. close to *C. tainanus*. More specimens would need to be tested to discount the possibility of *C. nielamensis* being capable of transmitting BTV. Few studies have reported on the prevalence of BTV in field populations of vectors [32–34], so it is difficult to establish when a species is not acting as a vector of BTV. However, the lower prevalence of BTV at high altitude, despite the higher proportion of *C. nielamensis* at these altitudes, suggests that *C. nielamensis* may not be acting as a vector, or at least not an efficient vector.

This study has shown that biting midge species capable of carrying BTV are present at 3300 m altitude sites in Shangri-La, which may explain the occurrence of BTV in yaks at that altitude. It is unknown if BTV moves seasonally to these highland meadows from nearby low altitude areas but as the seasonal movement of midges and viruses has been documented elsewhere [35], it is possible that this is also occurring in Yunnan or on the neighboring Tibetan Plateau. Based on the available evidence, any such movement could possibly be due to the dispersal of *Culicoides* sp. close to *C. obsoletus* which occurs at all altitudes studied here and has been shown to be infected with BTV; however, further work is needed to confirm that *C. nielamensis* or some other species is not also acting as a vector capable of moving virus between mid and high altitude sites.

Yaks are an important economic source for the Zang people who live in and around the Tibetan Plateau, and a recent study [36] indicated that the abortion rate of yaks in Qinghai Province, China, of 21.39% could be caused by BTV or other pathogens. Further study is needed to confirm the ability of BTV to cause clinical disease in yaks and subsequently to assist with disease control and prevention. Knowledge of the ecology of BTV in the extreme climate of Shangri-La may improve understanding of the effects of global warming on vector biology and disease epidemiology, particularly given the evidence that temperatures in the region are slowly rising.

## Conclusions

This study has identified three species of *Culicoides*, two of which appear to be new to science, which may be acting as vectors of BTV in high altitude areas of Yunnan. Further studies including laboratory infection and transmission of BTV is required to confirm the vector status of these species. The seroprevalence of BTV at high altitudes of Yunnan and the neighboring Tibetan Plateau could be caused by the seasonal movement of BTV vectors, such as *Culicoides* sp. close to *C. obsoletus*, from lower altitude areas where BTV is endemic.

## Data Availability

Data supporting the conclusions of this article are provided within the article. Raw data are available from the corresponding author upon request. The newly generated sequences were submitted to the GenBank database under the accession numbers MN513299–MN513328.

## Abbreviations

BTV: bluetongue virus
BLAST: basic local alignment search tool
BOLD: Barcode of Life Data system
*cox*1: cytochrome *c* oxidase subunit 1
Cq: quantification cycle
K2P: pairwise Kimura 2-parameter
NCBI: National Center for Biotechnology Information
NJ: neighbor-joining method
OIE: Office International des Epizooties
PCR: polymerase chain reaction
RT-PCR: reverse transcription PCR
RT-qPCR: reverse transcription quantitative PCR
